# Automated foveal location detection on spectral-domain optical coherence tomography in geographic atrophy patients

**DOI:** 10.1007/s00417-021-05520-6

**Published:** 2022-01-19

**Authors:** Andrea Montesel, Anthony Gigon, Agata Mosinska, Stefanos Apostolopoulos, Carlos Ciller, Sandro De Zanet, Irmela Mantel

**Affiliations:** 1 Department of Ophthalmology, Fondation Asile des Aveugles, Jules Gonin Eye Hospital, University of Lausanne, 15 Avenue de France, CP 5143, CH-1004 Lausanne, Switzerland; 2 RetinAI Medical AG, Bern, Switzerland

**Keywords:** Foveal location, Algorithm, Optical coherence tomography, Age-related macular degeneration, Geographic atrophy

## Abstract

**Purpose:**

To develop a fully automated algorithm for accurate detection of fovea location in atrophic age-related macular degeneration (AMD), based on spectral-domain optical coherence tomography (SD-OCT) scans.

**Methods:**

Image processing was conducted on a cohort of patients affected by geographic atrophy (GA). SD-OCT images (cube volume) from 55 eyes (51 patients) were extracted and processed with a layer segmentation algorithm to segment Ganglion Cell Layer (GCL) and Inner Plexiform Layer (IPL). Their en face thickness projection was convolved with a 2D Gaussian filter to find the global maximum, which corresponded to the detected fovea. The detection accuracy was evaluated by computing the distance between manual annotation and predicted location.

**Results:**

The mean total location error was 0.101±0.145mm; the mean error in horizontal and vertical en face axes was 0.064±0.140mm and 0.063±0.060mm, respectively. The mean error for foveal and extrafoveal retinal pigment epithelium and outer retinal atrophy (RORA) was 0.096±0.070mm and 0.107±0.212mm, respectively. Our method obtained a significantly smaller error than the fovea localization algorithm inbuilt in the OCT device (0.313±0.283mm, *p* <.001) or a method based on the thinnest central retinal thickness (0.843±1.221, *p* <.001). Significant outliers are depicted with the reliability score of the method.

**Conclusion:**

Despite retinal anatomical alterations related to GA, the presented algorithm was able to detect the foveal location on SD-OCT cubes with high reliability. Such an algorithm could be useful for studying structural-functional correlations in atrophic AMD and could have further applications in different retinal pathologies.

**Supplementary Information:**

The online version contains supplementary material available at 10.1007/s00417-021-05520-6.

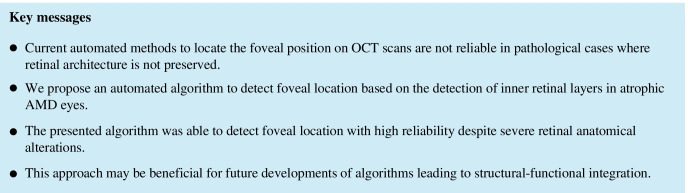

## Introduction

The fovea is the region of the retina with the highest density of cone photoreceptors, and it is responsible for sharp central vision [1]. As a result of its physiological development involving a centripetal displacement of outer retinal cells (whereas inner retinal cells are displaced centrifugally), it forms a central depression of the retinal surface [2]. Spectral-domain optical coherence tomography (SD-OCT) allows even further characterization of the foveal region, based on thickness difference between the different retinal layers, such as a thinning of the inner retinal layers, and a thickening of the outer retinal layers [3].

On optical coherence tomography of healthy eyes, the fovea is easily identifiable within the central macular region owing to its depression. At the same time, the recognition of retinal landmarks on OCT could be challenging in several retinal pathologies, either for anatomical alterations of the retinal architecture or for image artifacts [4, 5]. One such disease is age-related macular degeneration (AMD) with geographic atrophy (GA), characterized by atrophy of outer retinal tissue, retinal pigment epithelium and choriocapillaris [6, 7]. When atrophic lesions develop, their distance to the fovea is of particular interest, as the visual impact largely depends on the fovea-related location of the atrophy [8, 9].

Different approaches exist to locate the exact foveal position on SD-OCT in an automated fashion. Most methods rely on retinal thickness, defining the fovea as the point with the lowest distance between the internal limiting membrane and Bruch’s membrane [10, 11]. Such a definition, although shown to yield accurate results in healthy eyes, is not reliable in pathological cases where the retinal architecture is not preserved [12]. To our knowledge, there is currently no method of foveal detection in atrophic AMD eyes, and especially in GA patients, where retinal anatomy considerably deteriorates. We hereby propose an automated algorithm to detect foveal location based on SD-OCT volume scans and we report its performance in GA patients.

## Methods

### Study population and data recruitment

For image analysis and processing, SD-OCT scan volumes were randomly selected from an existing image data bank of the Medical Retina Department of our Institution. The inclusion criteria for the SD-OCT scans were the following: AMD with evidence of geographic atrophy on fundus autofluorescence and SD-OCT (Spectralis, Heidelberg Engineering, Germany), the availability of a SD-OCT macular map examination of at least 49 b-scans (6×6mm), and the absence of signs of exudations. The eyes with a history of retinal treatment, evidence of neovascular complication (past and present examinations), poor image quality, or confounding retinal pathologies were excluded. Adherence to the inclusion and exclusion criteria was confirmed by one senior retinal specialist (I.M.). This study was performed according to the ethical standards set by the Declaration of Helsinki and no informed consent was required in accordance with the ethics committee approval (CER-VD 2017-00493).

### Images analysis

The foveal detection analysis was conducted extracting the SD-OCT cube volumes from the SD-OCT machine and converting these to a coded .e2e file, after the removal of all personal patient data. Data was imported into the OmniViewer software (OmniViewer, RetinAI, Switzerland, software version 2019.4.0), a rating software designed specifically for the annotation and segmentation of images. In all the included SD-OCT volumes, the presence of the central foveal pit was identified and each scan was manually annotated by one of two experienced readers (A.M., A.G.) using a built-in point drawing tool. The readers indicated the foveolar a-scan by placing a point into the center of the fovea. The presence of the foveal depression, as well as the absence of inner retinal layers, and the relative thickness of the outer retinal layers were the evaluation criteria. In addition, the images were segmented for the atrophy location, using the most recent definition of retinal pigment epithelium (RPE) and outer retinal atrophy (RORA), defined as regions with signal hypertransmission beneath the retina, attenuation or complete disruption of the RPE, and photoreceptor disruptions, as evidenced by alterations of any of the layers from the ONL to the interdigitation zone [6]. Thus, the location of atrophy with regards to the fovea was visible allowing for differentiation between foveal or extrafoveal atrophy, depending if the RORA area included or not the foveal annotation.

### Algorithm development

The key observation used to design the fovea detection algorithm in the presence of GA is that the changes associated with this pathology influence mainly photoreceptors and RPE layers, and to a much lesser extent the inner retinal layers. Therefore, their appearance should not be significantly altered, making it possible to identify fovea in the en face projection. To this end, every OCT scan was processed to segment retinal layers using the previously described approach of Apostolopoulos et al. [13]. Prior to segmentation, the intensity of each input b-scan was normalized to the range (0.0 1.0) and the image was resized to half the resolution. After processing by the segmentation network, the resulting segmentation was upsampled to the original resolution.

Based on the resulting segmentation, the en face thickness map corresponding to GCL-IPL layers was computed and resampled to a common resolution of 0.025 × 0.025mm.

An inverted 2D Gaussian filter *G*(*x*, *y*) of size 81 × 81 pixels and *σ* (spread) of 7 pixels was hence constructed according to the formula:

$$G\left(x,y\right)=-\frac{1}{2\pi {\sigma}^2}{e}^{-\frac{-{x}^{2+{y}^2}}{2{\sigma}^2}}$$

**Fig. 1:**
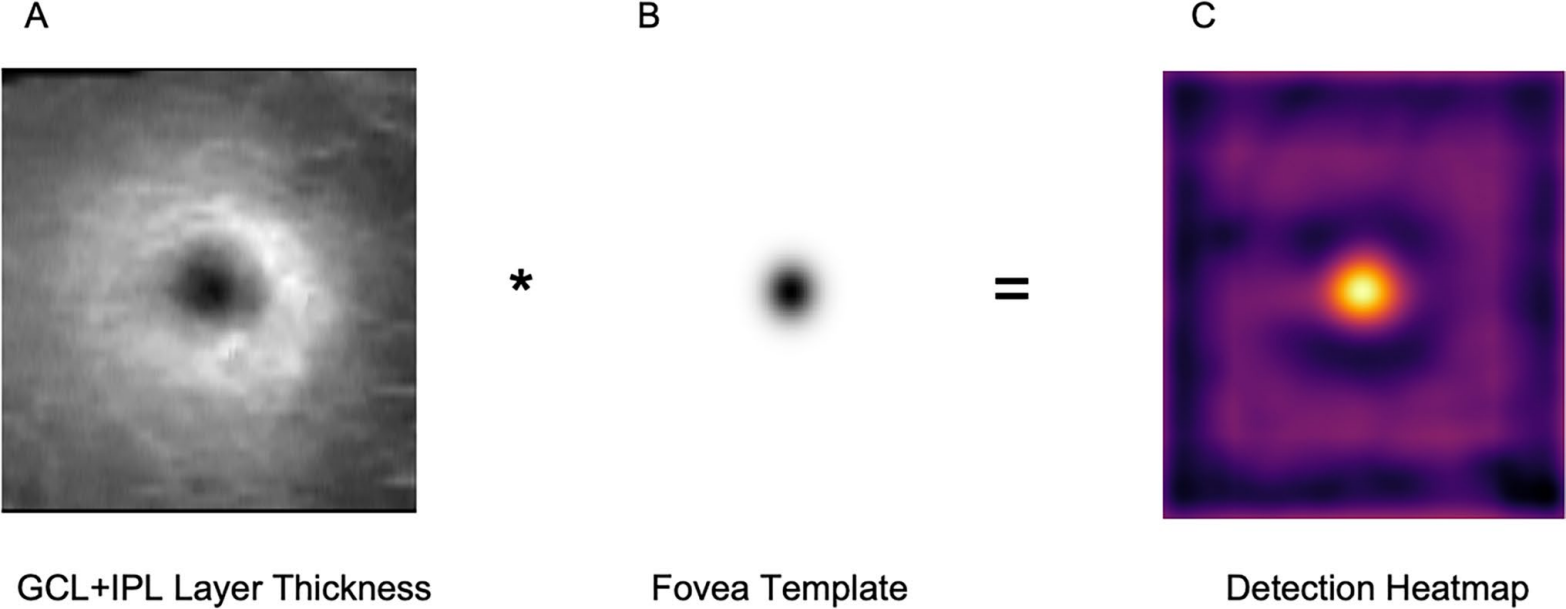
Algorithm working principle. Each SD-OCT scan was processed to obtain retinal layer segmentation and the en face thickness of GCL and IPL layers were computed (**A**). A fovea template corresponding to an inverted Gaussian filter was created (**B**). The template was convolved with the thickness map to find the best matching region (bright value in a resulting heatmap, **C**). The global maximum corresponds to the detected fovea. [SD-OCT, spectral-domain optical coherence tomography; GCL, ganglion cell layer; IPL, inner plexiform layer]

The *σ* value of the filter was chosen, so that 2*σ* corresponds approximately to foveola width i.e. 0.35mm (assuming a resolution of 0.025 × 0.025mm as described above). As seen in Fig. 1, the shape of the inverted Gaussian filter closely matched the pattern of the GCL-IPL thickness map around the fovea. For this reason, it was subsequently convolved with the resampled GCL-IPL thickness map. The global maximum position corresponded to the fovea location.

Formulation of our method also allows the introduction of a reliability score, which enables the identification of potentially large localization errors due to significant structural changes in the retina. The score corresponds to the numerical result of convolution of GCL-IPL thickness with the fovea template at the detected fovea location—the better the fit of the template to the detected region, the higher the score. Hence, low reliability score indicates that the en face thickness does not present a characteristic Gaussian pattern and the result may not be reliable.

### Outcomes and statistical analysis

We compared the performance of our algorithm for detecting the foveal center against the manual annotations. The detection was evaluated by computing the Euclidean distance between manual annotation and predicted location in en face projection. Outcome measures included the following: total detection error over all scans (mm, computed with respect to the grader), errors in vertical and horizontal en face axes (mm), errors for standard and dense scans (mm), and finally errors for foveal and extrafoveal RORA (mm). Additionally, we computed the detection error assuming the fovea at the center of the OCT scan, which can be considered the OCT device localization error, compared to the manual annotations. Another considered detection baseline was the thinnest point of the central retina. To this end, we computed total retinal thickness, and assigned fovea to the point with the smallest thickness. We excluded 50 pixels from the left and right sides of the b-scan, as the image borders often display acquisition artifacts. The device and retinal thickness detection errors were contrasted with the error computed using our method. A paired Student *t* test was used to compare different subgroups of errors (vertical vs horizontal, standard vs dense scans, foveal vs extrafoveal atrophy, and our method vs OCT device fovea estimation). In all analyses, *p* values < .05 were considered statistically significant.

## Results

A total of 55 eyes of 51 patients were included in the analysis. Characteristics of the study eyes are summarized in Table 1. Among the 55 eyes, 32 had foveal RORA and 23 extrafoveal RORA. Forty-two SD-OCT volumes were standard scans (6×6mm macular cube, 49 b-scans each), while 13 SD-OCT volumes were dense scans (6×6mm macular cube, 98 b-scans each). The mean RORA area was 7.42±5.06mm^2^.

**Table 1:** Characteristics of the study population

**Gender**	31.4% male (*n*=16) 68.6% female (*n*=35)
**Mean age**	82.3 ± 8.8 years
**Eyes** (right/left)	72% right eyes (*n*=40) 28% left eyes (*n*=15)
**RORA location**	58% eyes foveal RORA (*n*=32) 42% eyes extrafoveal RORA (*n*=23)
**RORA size** (mean)	Total RORA area, 7.42±5.06mm^2^ Foveal RORA area, 7.57±5.21mm^2^ Extrafoveal RORA area, 7.21 ±4.83mm^2^

*RORA*, retinal pigment epithelium and outer retinal atrophy

### Performance parameters

**Fig. 2:**
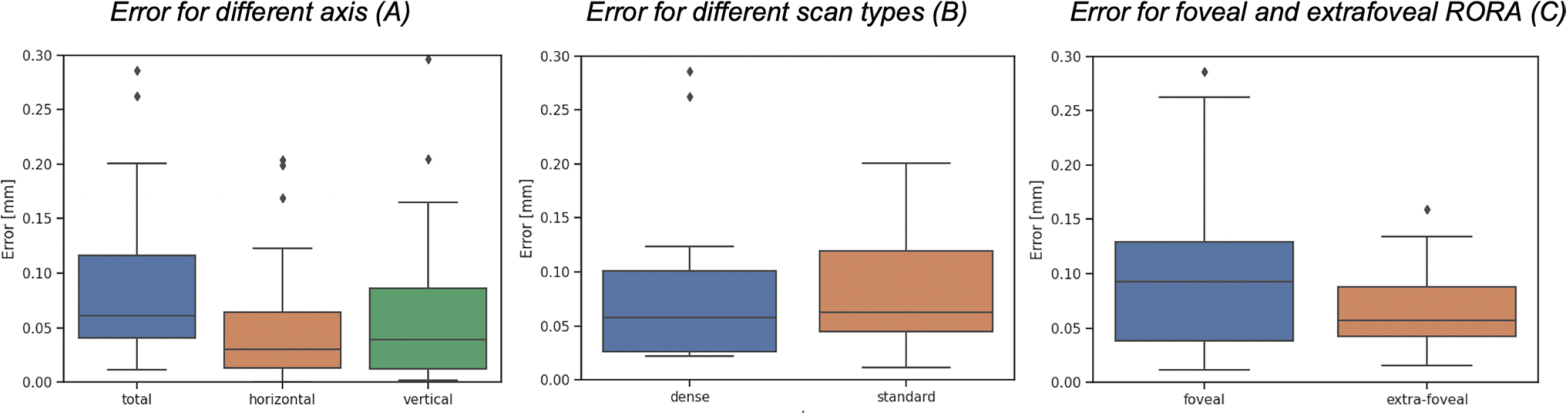
Fovea location performance. The three panels show respectively the distribution of errors for different axis (total, horizontal, and vertical) (**A**), the errors for dense and standard scans (**B**), and the errors for foveal and extrafoveal RORA (**C**). The point corresponding to the largest outlier of the total error of 1.07mm (Figure 6, extrafoveal atrophy, standard scan) is not shown in the plot for better readability). [RORA, retinal pigment epithelium and outer retinal atrophy]

**Fig. 3:**
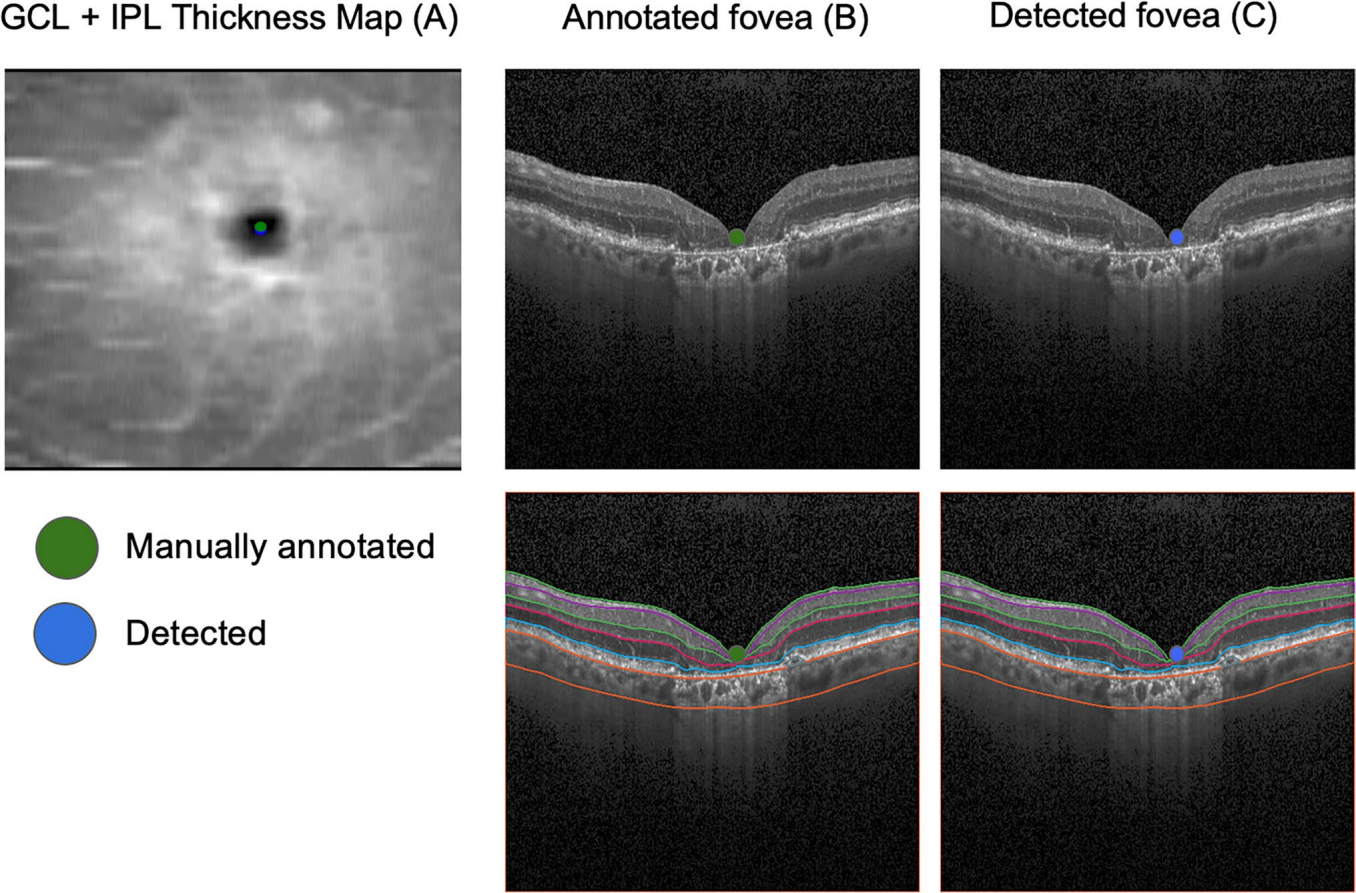
Qualitative analysis of the results. The figures illustrate an example of high correspondence between manual (green dot) and detected (blue dot) foveal location. On the left panel (**A**), it is possible to appreciate the two foveal localizations on the GCL-IPL thickness map (0.049 mm error). Manual foveal annotation on the SD-OCT standard b-scan (**B**, above) and the corresponding layer segmentation (**B**, below) and detected foveal annotation on the SD-OCT standard b-scan (**C**, above) and the corresponding layer segmentation (**C**, below). [GCL, ganglion cell layer; IPL, inner plexiform layer; SD-OCT, spectral-domain optical coherence tomography]

**Fig. 4:**
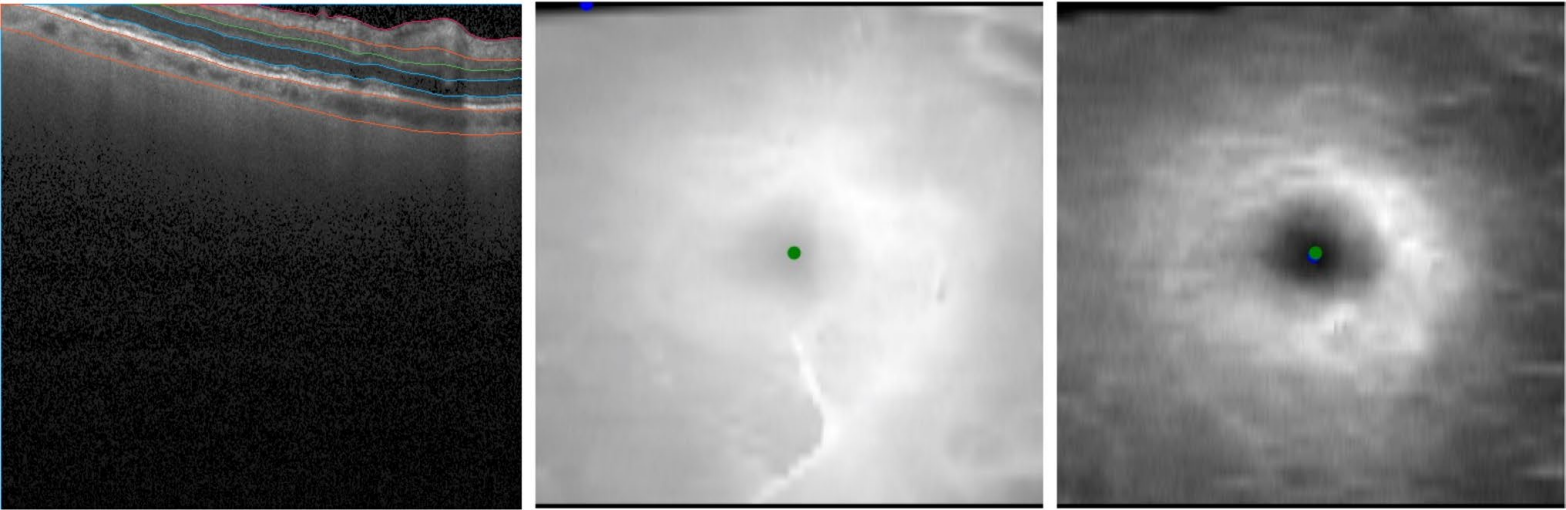
Foveal detection in the presence of acquisition artifacts. Left: shifted b-scan causes partial occlusion of the retinal layers; middle: this results in lower total retinal thickness in the occluded region and large localization error of the method based on thinnest point of the retina (blue dot) in comparison to the manual ground-truth (green dot); right: the proposed method considers a large point neighborhood in the image to detect fovea (blue dot) and as a result it is not affected by shifting acquisition artifacts

The mean total localization error was 0.101±0.145mm and the mean error in horizontal and vertical en face axes was 0.064±0.140mm and 0.063±0.060mm (*p*=.9258), respectively. The mean error for standard and dense scans was 0.104±0.159mm and 0.090±0.089mm (*p*=.7629), respectively. The mean error for foveal and extrafoveal RORA was 0.096±0.070mm and 0.107±0.212mm (*p*=.7730), respectively (Table 2). The error obtained if the center of the scan was taken as fovea resulted in an error of 0.313±0.283mm and was significantly larger than the error obtained with our method (*p*<.001). Similarly, a standard technique of assigning fovea to the thinnest part of the retina performed significantly worse with the average error of 0.843±1.221 (*p*<.001). A graphical analysis of the distribution of the errors was performed (Fig. 2). Visual examples of predicted and annotated fovea locations are shown in Figs. 3 and 4.

**Table 2:** Summary of the results

	Mean error [mm±SD]	Error comparison^†^
**Horizontal axis**	0.064±0.140	*p* = .9258
**Vertical axis**	0.063±0.060	
**Standard scans**	0.104±0.159	*p* = .7629
**Dense scans**	0.090±0.089	
**Foveal RORA**	0.096±0.070	*p* = .7730
**Extrafoveal RORA**	0.107±0.212	
**Total**	0.101±0.145	*p* <.001^*^
**Assuming center of the scan**	0.313±0.283	
**Assuming the thinnest central retinal point**	0.843±1.221	*p* <.001^*^

*SD*, standard deviation; ^†^paired *t* test; ^*^statistically significant; *RORA*, retinal pigment epithelium and outer retinal atrophy

## Discussion

The algorithm proposed in this study was able to detect the foveal location in atrophic AMD with high reliability, based on SD-OCT volume scans. To our knowledge, there is no report about an algorithm able to detect foveal center on atrophic eyes. However, this could be beneficial for accurate and reproducible structure-function correlation and follow-up studies. GA can cause severe deformation of the fovea, thus presenting a major challenge in automated detection [14].

Only a few methods have been reported to focus on automated foveal detection, and most of them were done in healthy eyes. Niu et al. have proposed an automated foveal detection algorithm using the topological concept of saliency, to account for the foveal pit as a whole three-dimensional object, and not only a single distance on a single b-scan [15]. Other works have shown reliable results using inner retinal layers thickness to assess foveal location in healthy eyes [16–18]. These approaches have the advantage of using more parameters compared to retinal thickness as a whole, applying more direct structural knowledge of normal foveal anatomy. However, to our knowledge, none of these methods has been tested on atrophic AMD eyes.

Our presented algorithm is based on the detection of inner retinal layers that have been shown to remain intact in the majority of GA eyes, or at least to be affected by the degenerative process much later than outer retinal layers [19–21]. The slower anatomical deterioration of inner retinal layers may be due to lower vulnerability and little dependency on an intact RPE [21].

In our investigation, foveal location was detected with a high level of accuracy, closely matching the manual annotation of foveal center in OCT images among GA patients (Fig. 3). Foveal detection accuracy was comparable between dense OCT scans and standard scans, (*p* =.7629), meaning that the greater axial resolution provided by the first ones did not result in a better detection ability. It is already established that an increase in the scanning density of SD-OCT acquisitions is associated with more reliable measurements of retinal thickness, especially in pathological eyes [22, 23]. In our case, the higher scanning density did not improve the accuracy of the detection, and this could be due to an already satisfactory grade of efficiency provided by standard scans resolution and resampling en face projections to the same resolution. Indeed, thanks to resampling the en face thickness map to higher resolution in vertical direction and modeling the foveal pit as a 2D Gaussian filter, it is possible to potentially identify foveal localization with sub-b-scan resolution accuracy.

Remarkably, no statistically significant difference was reported comparing foveal and extrafoveal RORA. This could be explained by the relative preservation of GCL and IPL layers in GA eyes, enabling a reliable foveal detection also in presence of foveal atrophy [20, 24]. Furthermore, the presence of RPE debris of underlying drusen does not affect the performance, as only the GCL-IPL thickness map is considered during processing (Fig. SI 1). While architectural changes induced by outer retinal atrophy could have affected the reliability of IPL-INL boundary delineation and subsequent fovea detection, we observed that it was not the case in our dataset (Fig. SI 2).

**Fig. 5:**
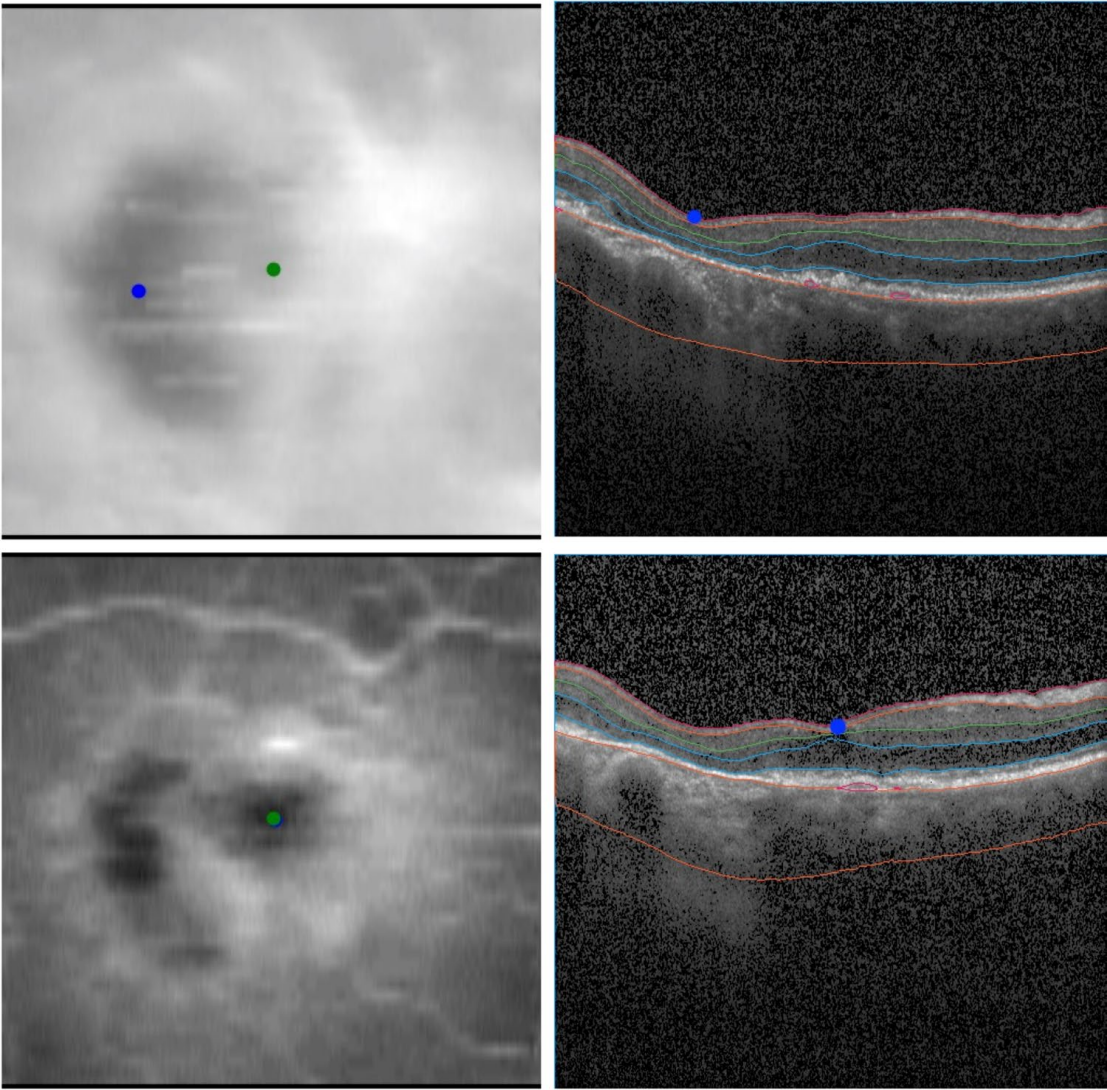
Foveal detection in the presence of advanced GA. Top: advanced GA results in retinal thinning and affects methods that rely on minimum retinal thickness (blue dot), which shows large disagreement with the manual annotation (green dot); bottom: the proposed method relies only on GCL-IPL thickness, which is not affected by GA to a large extent. Hence, the automatic detection (blue point) shows good agreement with ground-truth (green dot). [GA, geographic atrophy; GCL, ganglion cell layer; IPL, inner plexiform layer]

In addition, the proposed method showed a considerably smaller detection error than the detection error assuming fovea at the center of the scan, which is how the algorithms inbuilt in OCT devices center the scans at the fovea by means of the patient’s fixation. Another considered baseline, which assumes fovea at the thinnest point of the retina, also performed worse than our method. Despite excluding points at the border, in several cases, the upper part of the retina image was partly cut at the top due to movement artifact, resulting in lower retinal thickness at this point and ultimately false fovea detection (Fig. 4). Relatively large errors were observed in cases of advanced GA, where significant degradation of photoreceptors and RPE layer significantly impacted the retinal thickness (Fig. 5).

Our approach is more robust to the imaging artifacts due to the convolution with Gaussian kernel, which considers properties of a large image neighborhood to detect fovea, instead of a single point value, as is the case for the standard techniques. Our method performs better also in cases of advanced GA, as it relies on GCL and IPL layers, which are affected by GA to a lesser extent.

Thus, possible applications of the proposed algorithm include the possibility of more precise placement of measurements such as Early Treatment Diabetic Retinopathy Study (ETDRS) grid delineation on the OCT volume. In addition, further application of this algorithm could be studying structural-functional correlations in atrophic AMD, as previously described in visual fields or microperimetry studies, aiming for a better understanding of the impact of disease-specific morphological changes on visual function [25, 26]. The proposed method could be a useful tool for future studies that integrate the functional consequences into the evaluation of atrophic AMD changes, which could be of particular interest as the visual function of GA patients largely depends on the fovea-related location of the atrophy [8, 9]. Furthermore, the presented method could be applied for retinal pathologies other than atrophic AMD, for which the pattern GCL-IPL layers thickness is not drastically changed, such as central serous chorioretinopathy (CSC) [27].

**Fig. 6:**
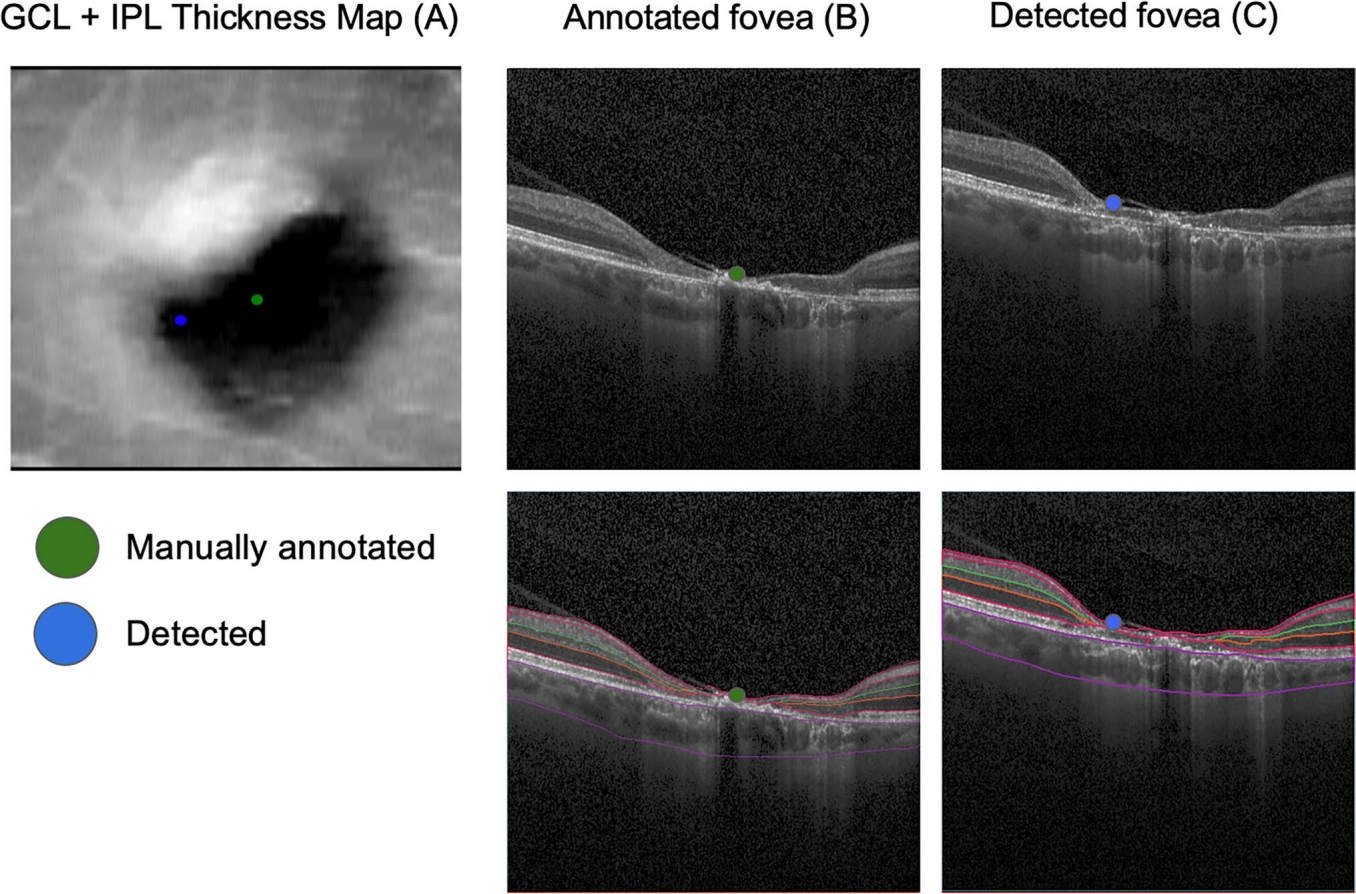
Example of prominent failure (outlier case). The figure illustrates a case of prominent failure defined as an outlier. The left panel (**A**) shows the manual (green dot) and detected (blue dot) foveal location on the GCL-IPL thickness map (1.07 mm error). Manual foveal annotation on the SD-OCT standard b-scan (**B**, above) and the corresponding layer segmentation (**B**, below). Detected foveal annotation (**C**, above) and the corresponding layer segmentation (**C**, below). Significant degeneration of all retinal layers, including GCL-IPL layers, resulted in distorted en face projection, which no longer matched the Gaussian profile. This resulted in inaccurate detection. [GCL, ganglion cell layer; IPL, inner plexiform layer; SD-OCT, spectral-domain optical coherence tomography]

**Fig. 7:**
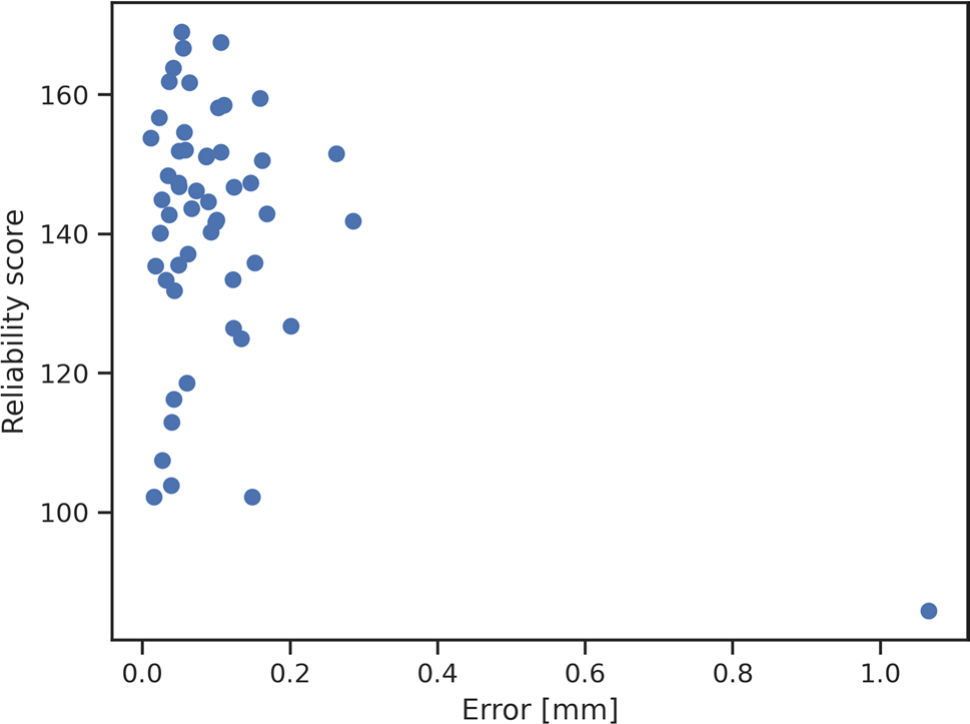
Distribution of reliability scores. Reliability score allows for identification of cases, where GCL-IPL en face thickness map does not present the characteristic Gaussian profile. Indeed, the localization failure presented in Figure 6 is shown here as an outlier on the right with a lower reliability score. [GCL, ganglion cell layer; IPL, inner plexiform layer]

While the quantitative results indicated high mean accuracy of the algorithm, the graphic analysis of the distribution of the errors (Fig. 2) revealed the presence of some relevant outliers. Closer inspection of those examples, including the most prominent outlier with a total estimation error of 1.07 mm (Fig. 6), revealed that all retinal layers showed significant degeneration within a large region. This resulted in GCL-IPL en face projection not presenting a characteristic Gaussian pattern, which is essential for our method to accurately detect fovea. Indeed, the requirement for GCL-IPL layers to remain relatively intact is the major limitation of our method. The mean error was evidently influenced by these outliers, and the distribution of all errors is shown in Fig. 2. However, this limitation can be alleviated by the introduction of a reliability score (Fig. 7), which indeed was significantly lower for the large localization error seen in Fig. 6. Detection examples of the remaining two outliers seen in Fig. 2 are shown in Fig. SI 3.

In summary, the current study reports the application of a novel automated algorithm for foveal automated detection on SD-OCT scan. Our approach showed promising results comparable to the manual annotation of experienced human readers.

Another limitation of our analysis is that our model was validated on a relatively small size of the sample. The next step in validating the performance of the algorithm would be to increase the study cohort and add OCT scans acquired with OCT devices other than Heidelberg Spectralis. Further research will also be needed to improve the segmentation algorithm. Furthermore, in the present study, the algorithm was only tested on non-exudative GA patients. An extension to other outer retinal pathologies, such as neovascular AMD or CSC, would need validation in order to reach a more global validation of the algorithm.

In conclusion, the presented algorithm was able to detect the foveal location on SD-OCT cubes with high reliability, despite retinal anatomical alterations related to geographic atrophy. A variety of possible applications with respect to the functional importance of the fovea can be considered, including structural-functional correlations in atrophic AMD. An extension to various outer retinal pathologies could be considered, requiring scientific validation.

## Supplementary Information

Fig. SI 1 Foveal detection in the presence of RPE debris. Left: example of detection accuracy (blue dot—automatic method, green dot—manual annotation) in case of existing RPE debris visible in the b-scan (left). Our method is not affected by it, as it relies only on GCL-IPL thickness profile. [RPE, retinal pigment epithelium; GCL, ganglion cell layer; IPL, inner plexiform layer] (PNG 11487 kb)

High Resolution (TIFF 12656 kb)

Fig. SI 2 Examples of layer delineation in the presence of Outer Retinal Atrophy (ORA). IPL-INL border delineation (pink line) may suffer in the presence of ORA if such cases were not considered during development of the segmentation model. This could further impact fovea detection, which utilizes GCL-IPL thickness map. However, we did not observe it in our dataset and changes in the retinal structure did not affect the accuracy of IPL-INL border detection. [IPL, inner plexiform layer; INL, inner nuclear layer; GCL, ganglion cell layer; ORA, outer retinal atrophy] (PNG 3909 kb)

High Resolution (TIFF 24792 kb)

Fig. SI 3 Examples of larger disagreements with ground-truth. Automatic fovea detection (blue) and ground-truth (green) overlaid on the GCL-IPL thickness map. The examples correspond to the remaining two error outliers shown in Figure 2 (0.28mm and 0.26 mm error). [GCL, ganglion cell layer; IPL, inner plexiform layer] (PNG 41895 kb)

High Resolution (TIFF 12726 kb)

## Data Availability

The data that support the findings of this study are available from the corresponding author upon reasonable request.
