# Supplementary figures and images for: Automated foveal location detection on spectral-domain optical coherence tomography in geographic atrophy patients
Source: Graefes Arch Clin Exp Ophthalmol. 2022 Jan 19;260(7):2261–70. doi: 10.1007/s00417-021-05520-6 (PMC9203415; doi:10.1007/s00417-021-05520-6)

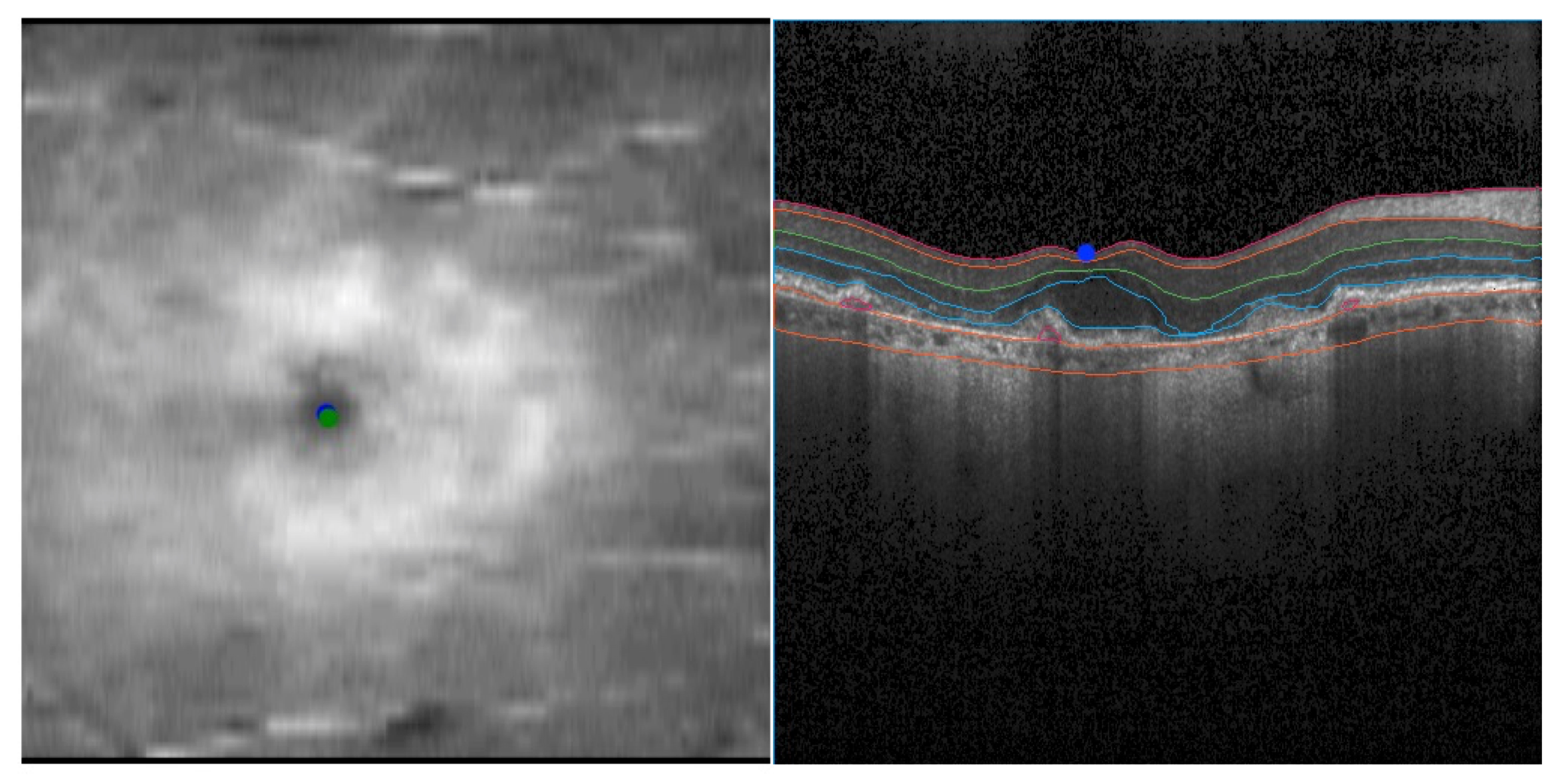

Supplement: Supplementary file 1 — Foveal detection in the presence of RPE debris. Left: example of detection accuracy (blue dot—automatic method, green dot—manual annotation) in case of existing RPE debris visible in the b-scan (left). Our method is not affected by it, as it relies only on GCL-IPL thickness profile. [RPE, retinal pigment epithelium; GCL, ganglion cell layer; IPL, inner plexiform layer] (PNG 11487 kb) [file 417_2021_5520_Fig8_ESM.png]

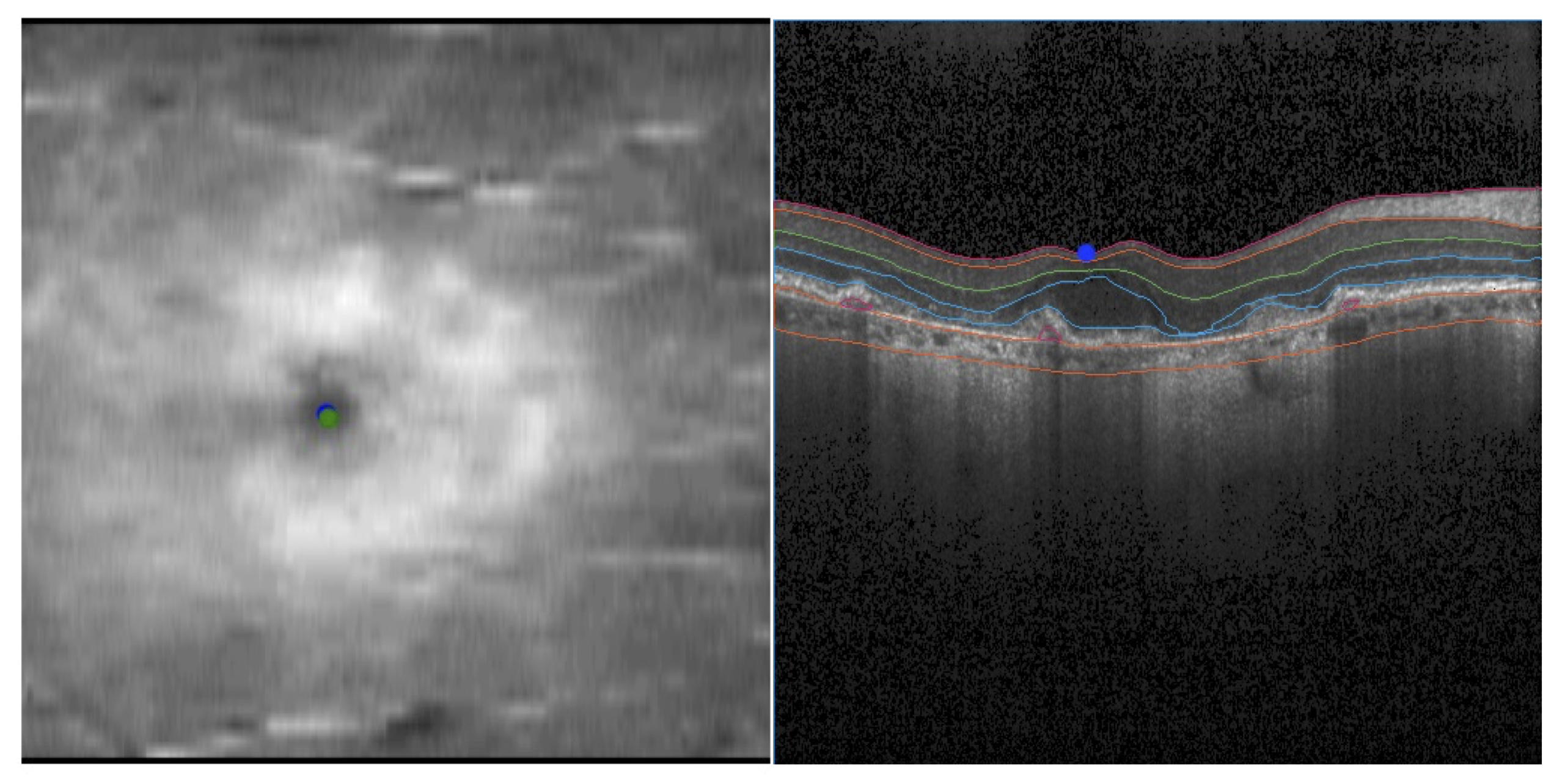

Supplement: Supplementary file 2 — High Resolution (TIFF 12656 kb) [file 417_2021_5520_MOESM1_ESM.tiff]

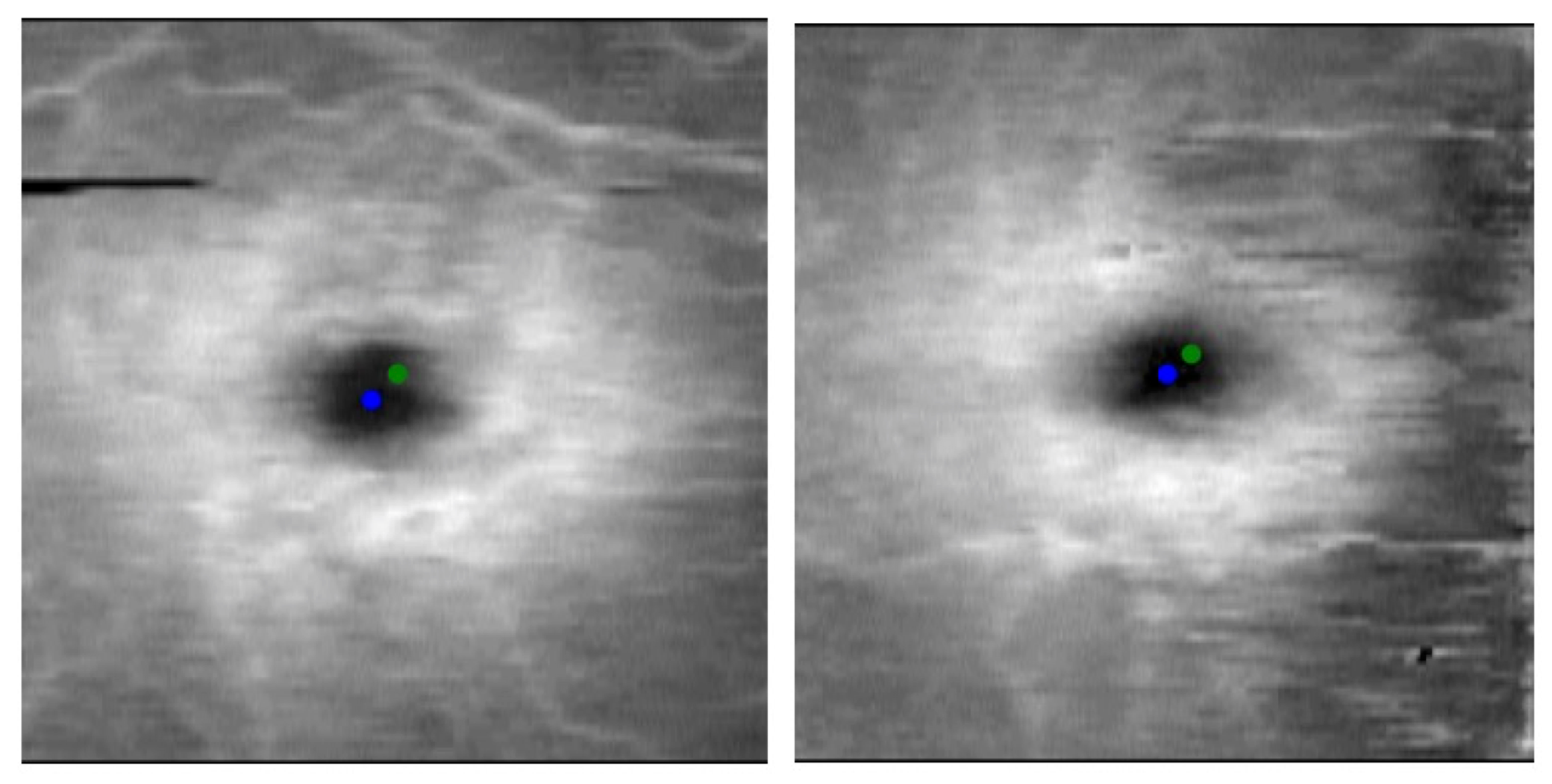

Supplement: Supplementary file 3 — Examples of layer delineation in the presence of Outer Retinal Atrophy (ORA). IPL-INL border delineation (pink line) may suffer in the presence of ORA if such cases were not considered during development of the segmentation model. This could further impact fovea detection, which utilizes GCL-IPL thickness map. However, we did not observe it in our dataset and changes in the retinal structure did not affect the accuracy of IPL-INL border detection. [IPL, inner plexiform layer; INL, inner nuclear layer; GCL, ganglion cell layer; ORA, outer retinal atrophy] (PNG 3909 kb) [file 417_2021_5520_Fig9_ESM.png]

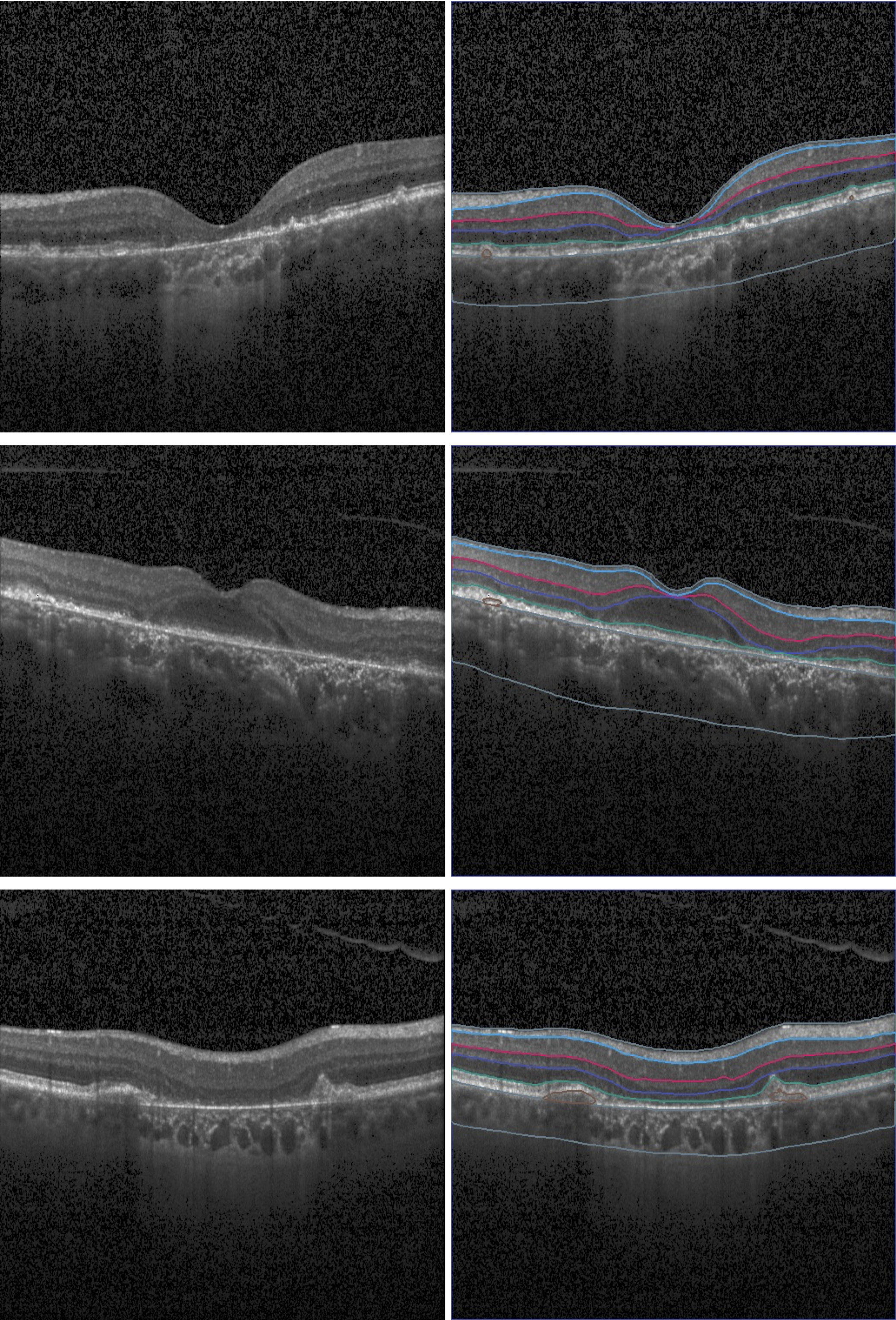

Supplement: Supplementary file 4 — High Resolution (TIFF 24792 kb) [file 417_2021_5520_MOESM2_ESM.tiff]

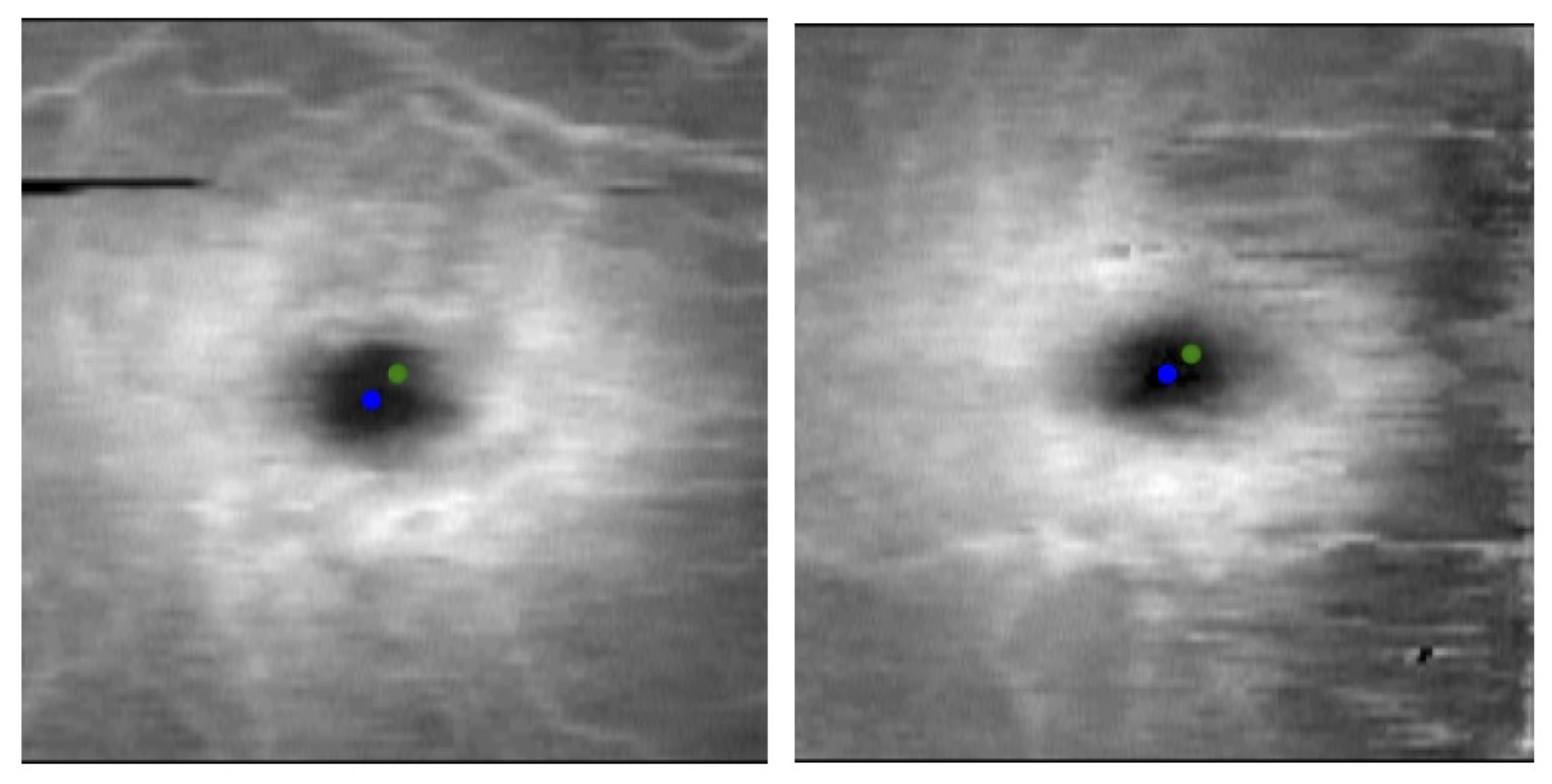

Supplement: Supplementary file 6 — High Resolution (TIFF 12726 kb) [file 417_2021_5520_MOESM3_ESM.tiff]
